# The hubris and humility of cancer pharmacology in the post immuno‐oncology era

**DOI:** 10.1002/prp2.527

**Published:** 2019-10-08

**Authors:** John S. Lazo

**Affiliations:** ^1^ Departments of Pharmacology and Chemistry University of Virginia Charlottesville VA USA

**Keywords:** cancer, cancer cell lines, drug discovery, immune therapy, preclinical mouse models

## Abstract

Cancer is a dreaded word, which has stimulated monumental efforts to discover and deliver effective cancer treatments for more than half a century. During the past two decades, our understanding of the molecular pathogenesis of cancer has increased remarkably. This has fostered an explosion in the number of experimental agents and clinical trials coupled with a dramatic rise in the regulatory approval of therapies for human cancers. Unfortunately, our preclinical models perform poorly as predictive platforms for the ultimate success of clinical candidates, reflecting the complexity of cancer. Moreover the common combination of cancer drugs prescribes the need for a better understanding of the fundamental pharmacology of each agent. Here I briefly outline some of the fundamental changes that have and have not occurred in cancer pharmacology during the past two decades and prognosticate on possible future directions.

**Abbreviations**
BiTE: bispecific T cell engager
CCLE: Cancer Cell Line Encyclopedia
EGFR: epidermal growth factor receptor
EMA: European Medicine Agency
FDA: Food and Drug Administration
GDSC: Genomics of Drug Sensitivity in Cancer
HGF: hepatocyte growth factor
NCI60: National Cancer Institute 60 cell lines
PARP: poly(ADP‐ribose) polymerase
PROTAC: proteolysis‐targeting chimera
TCGA: Tumor Cell Genome Atlas
VEGF: vascular endothelial growth factor

## 1 INTRODUCTION

**Figure 1:**
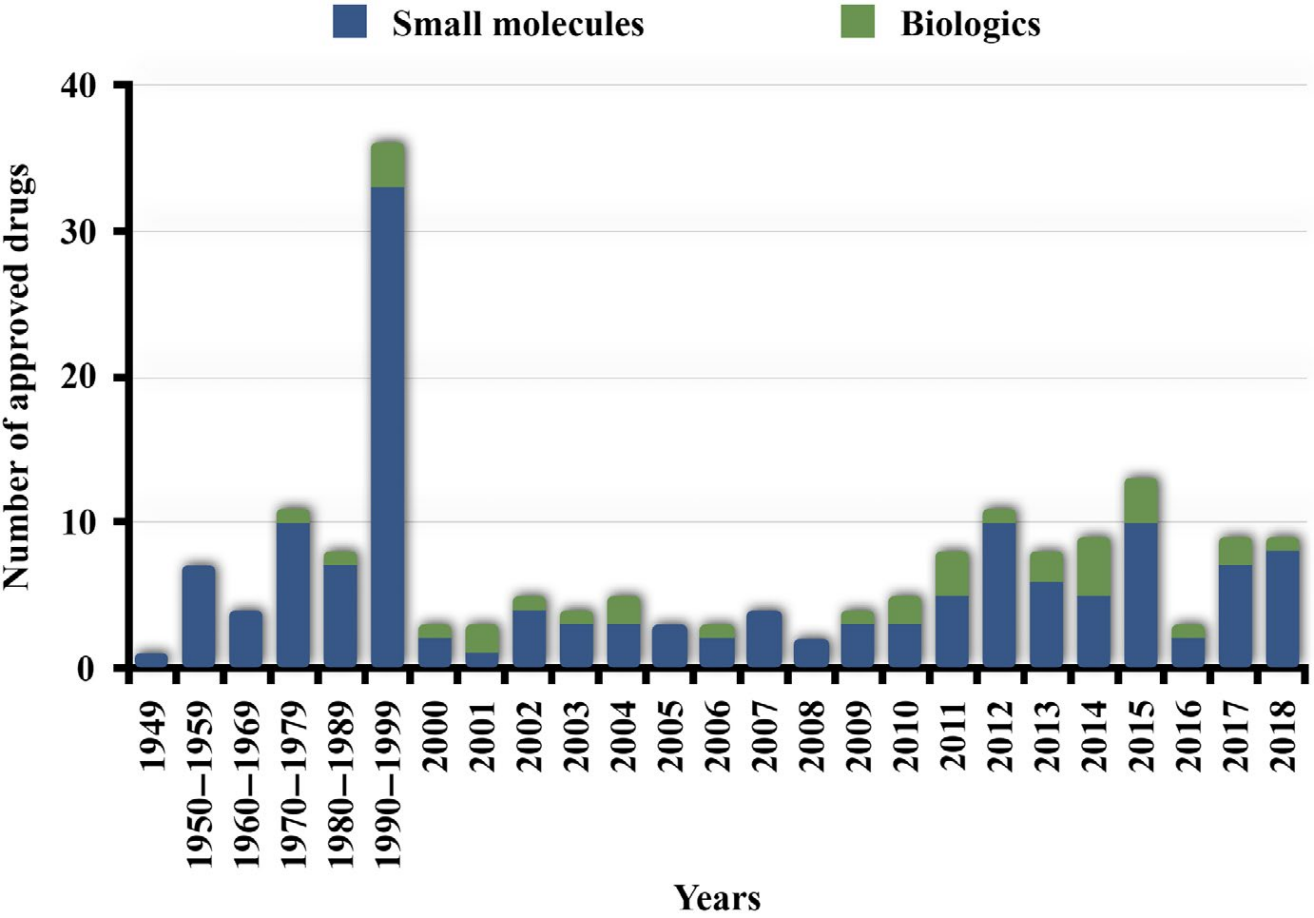
Number of FDA approved drugs for cancer. Included in the biologics are enzymes, protein‐based agents, including antibodies, and cellular therapies. Created with information from Drugs@FDA and 9

Hardly a month passes without some announcement in the lay press about a new promising approach to detect or treat human cancer. The sad fact is that ~9.5 million individuals worldwide will die of cancer this year.^1^, ^2^ Almost two decades ago, Professor John A. Hickman and I co‐authored an overview of the changes and challenges for cancer pharmacology in the post‐imatinib era.^3^ It was a somewhat heady time when, armed with the recent sequencing of the human genome, many thought we were beginning to understand at least a few of the fundamental molecular causes of human cancer. We had small molecules targeting oncogenic tyrosine kinases being approved by the FDA (Figure 1). The first chimeric monoclonal antibody, rituximab, had been approved by regulatory agencies in 1997‐1998 followed by trastuzumab. In 2000, the six hallmarks of cancer were declared,^4^ which helped stimulate the discovery and testing of many small molecules and biologics.

Much has changed since then, however. At the time our overview was written, cancer drugs were largely cytotoxic agents designed to kill rapidly dividing cells and they were a small fraction of the pharmacopeia and of most companies’ portfolio. Drug combinations were formulated largely by empirical tests that employed drugs with different mechanisms of action, avoiding overlapping toxicities and common mechanisms of resistance. Now, the US Food and Drug Administration (FDA)‐ and the European Medicine Agency (EMA)‐approved cancer therapies number in the hundreds (Figure 1); imatinib can reasonably claim to be the father or grandfather to 35 FDA‐approved tyrosine kinase inhibitors with many more in clinical development. Targeted drugs have assumed a major role in cancer regimens and have been one driver for the development of more personalized treatment schedules combining diagnostics with therapeutics. Some individuals have thoughtfully challenged the ultimate efficacy of the new targeted therapies and the personalized medicine approach,^5^, ^6^ while others have demurred proposing that molecular characterization methodologies will evolve and that improved treatments are likely to yield better results in the future.^7^, ^8^ It is my view that cancer pharmacologists, in general, have an optimistic disposition and fall into the second school of thought, perhaps because their research addresses such a challenging disease. Many of the targeted therapies are small molecules but there has been a steady growth in the role of biologics, which could even overtake small molecules in the future^9^ (Figure 1). Two decades ago, it was hoped that gene therapy or at least nucleic acid‐based treatments would be widely adopted but this has not developed as anticipated. Currently, the emergence of durable responses in some patients with cancers with agents that modify the immune system using antibodies, small molecules and even cellular therapy (including cells that have been genetically modified) has generated enormous, possibly excessive, enthusiasm. Despite the adoption and enthusiasm for targeted and immune therapy for cancer, much also has not changed. The cytotoxic agents, including radiation, continue to be the foundation of most cancer treatments. My guess is that the cytotoxic agents are not likely to depart in the near future. This raises the question: are we over hyping our successes or should we be humbler because we still have little insight into why some cancer agents are effective and others are not? The immediate hope that personalized medicine strategies would radically alter the patient outcomes have sadly not yet been fully achieved.^5^ It may even have regrettably redirected valuable funds to misguided missions. So perhaps it is timely to reflect on at least a few of those previous thoughts^3^ and consider the new challenges for the field of cancer pharmacology?

### 1.1 The complexity of cancer

Long ago, we appreciated that cancer was not a single disease and focused our treatments based on the organ from which the tumor arose and its histological features. In the past two decades, with advanced tools, we have uncovered a multitude of molecular, biochemical and cellular differences among and within tumors, which have exposed a vast number of potential therapeutic targets, some druggable, others not.^10^ Some of the putative targets may have essential normal functions, which may or may not predict toxicity, if subjected to target engagement.^11^ The real challenge has been to rapidly and robustly prioritize, which of these differences are critical for the disease at the time of diagnosis. For example, we have exploited the availability of high throughput sequencing instrumentation to map, ever so deeply, the DNA mutations, amplifications, and deletions present in human cancer genomes. DNA mutations are abundant and in some cases the mutations themselves generated cells that spontaneously create more mutations because of their inability to control the fidelity of DNA replication and repair. The genetic profiles of human tumors have produced entirely new classification schemes that have been proposed to reflect disease prognosis and therapeutic response.^12^ At our institution, like many others, we now have regular Molecular Tumor Board meetings to supplement the more traditional histologically‐aligned tumor boards, such as Breast Cancer Tumor Boards. Unfortunately, many of the observed DNA mutations have no known function, are not pharmacologically actionable, or may be legacies of prior events with only a “passenger” role. Some tumor types have no obvious generalized genetic driver mutations, such as ovarian cancer, or are mutationally “silent”, such as many pediatric cancers. This complexity along with the recognition that there are many nongenetic factors that cause or promote cancer growth or dissemination has propelled the adoption of other analytical methodological approaches, collectively termed “omics”, to decode the fundamental drivers of cancer, including transcriptional analysis with RNA sequencing, epigenetic expression profiling, and mass spectrometry‐based proteomic and metabolic profiling, to mention just a few strategies being applied to primary tumors, metastases, and even circulating tumor cells. The results of these big data enterprises are widely available in public databases, such as the Tumor Cell Genome Atlas (TCGA), and are frequently used in drug discovery programs. Differences in gene expression between the primary tumor and metastases or even within a single tumor further adds to the complexity of cancer. This heterogeneity has propelled the use of multidimensional, high‐content, single cell analyses to explore cancer cell sensitivity to drugs. We also now more deeply appreciate how important the tumor microenvironment, such as pH, oxygenation, glucose and amino acid availability, inflammation, and stromal cell content, is in determining how tumors react to compounds. Tumors rapidly adapt to treatments leading to drug resistance. We recognize that the extracellular matrix and rigidity in culture can markedly alter response to compound exposure. This has fostered efforts to replace traditional two‐dimensional culturing platforms with three‐dimensional ones^13^ and the development of organoid systems for preclinical studies.^14^ It is surprising, however, that no single in vitro platform has emerged as optimal to predict drug responsiveness.

Two decades ago, the most advanced collection of human tumor cell lines to study potential therapies was the National Cancer Institute's 60 cell lines, known as the NCI60.^15^ The hope was that this in vitro panel would empower users to identify which tumor type would respond to a given treatment in vivo. Ultimately and unfortunately, this hypothesis was not supported by the data. Some investigators concluded a bigger tumor panel was needed, embracing the “bigger is better” philosophy, so that today there are human tumor cell collections, such as the Cancer Cell Line Encyclopedia (CCLE) and the Genomics of Drug Sensitivity in Cancer (GDSC), which comprise > 1000 cell lines and have been highly annotated for compound responsiveness as well as DNA mutational status, gene expression, and proteomic profiles. Obviously, maintaining such large cell line sets, even with the increased availability of automated liquid handling platforms, is labor‐intensive and expensive, especially at a time when research funds are finite. The optimal cell panel size remains to be tested rigorously. The availability of methods to genetically modify cancer cells with CRISPR technology, along with more transient suppression methods, such as small interfering RNA, short hairpin RNA and microRNA, has exposed the importance of the cellular context in cancers. We also know that some cancers have stem cell‐like populations that are likely to have the major role in tumor growth but are intrinsically resistant to most therapies.^16^ *Caveat emptor*: one cell line does not a conclusion make.

**Figure 2:**
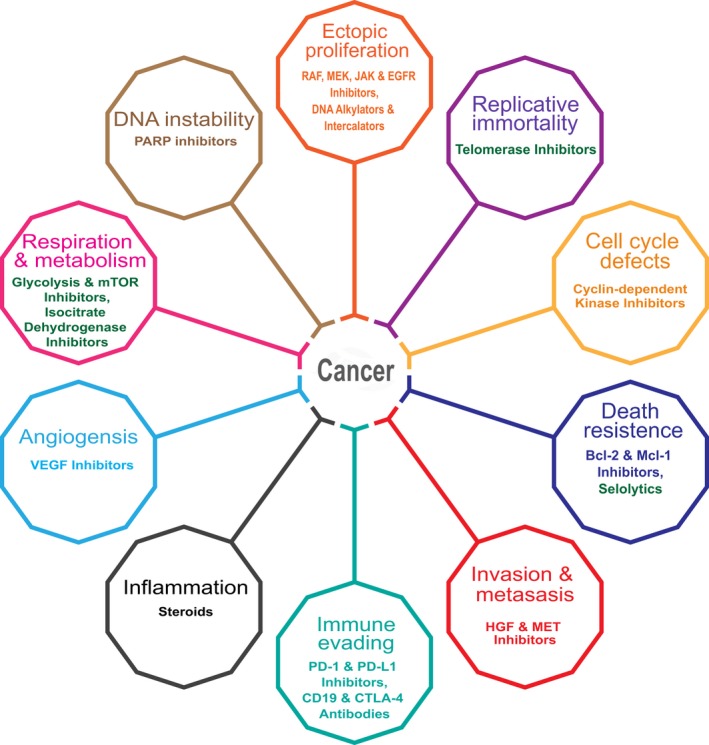
The decagon of cancer attributes. This diagram illustrates the major characteristics associated with cancer and a few selected classes of pharmacological agents used to perturb the processes. Classes of experimental agents that have not received regulatory approval are noted in dark green

Preclinically, mouse models have dominated as the test platforms to evaluate new therapies sharing similar complexities and questionable predictive value with in vitro models. Tumors are now recognized by many as dysfunctional organs with an essential dependency on stromal cells, including endothelial, fibroblasts, pericytes, macrophages and other immune cells.^4^, ^17^, ^18^ Robust debate continues about the utility of cell line‐derived, genetically engineered, environmentally‐induced, or patient‐derived mouse tumor models.^19^ How important is the location of the tumor implantation; should it be subcutaneous, intraperitoneal, or orthotopic? Several reviews summarizing the repertoire of available murine models have been published and the reader is directed to them for further details.^19^, ^20^, ^21^, ^22^ The selection of route of compound administration also needs to be considers: should it be oral or intravenous or will intraperitoneal or subcutaneous suffice? In addition, cancer drugs are rarely administered as single agents so the schedule of drug treatments adds another level of complexity. In summary, there also has been no consolidation on the appropriate preclinical in vivo models.^19^ *Caveat emptor*: one tumor model does not a conclusion make. Collectively, therefore, it is safe to state the field of cancer pharmacology has grown even more complex in the past two decades. This notion is reflected in the evolution of the hallmarks of cancer,^4^, ^17^, ^18^ which have been expanded to a decagon of attributes (Figure 2) and will likely grow.

### 1.2 Tumor addiction to oncogenes

The prevailing belief two decades ago was the only good tumor cell was a dead one. The community began to identify oncoproteins that one could document, largely by genetic means, were responsible for cancer cell proliferation and invasion. Many of these oncoproteins and even oncometabolites, like 2‐hydroxygluterate, formed the foundation of the cancer hallmarks (Figure 2). Often the oncoproteins exhibited a gain of function activity, such as with an oncogenic tyrosine kinase, that could be targeted with small molecules or antibodies. In some cases, however, it was bewildering how inhibiting the newly gained function could cause cancer cell death. The notion emerged that tumor cells became dependent or “addicted” to the new enzyme activity, perhaps by re‐wiring signaling pathways, and inhibition or withdrawal of the enzyme activity would prove lethal. This “oncogene addiction” hypothesis was coupled with the observation that cancer cells could die not only by the seemingly passive and somewhat messy process of necrosis but also by the more processive and seemingly regulated program termed apoptosis. Proteins were identified that enabled tumors to progress by enhancing survival and prevented drug‐induced apoptosis.^23^ Landmark BH3‐mimetic drugs, such as ABT‐199 or venetoclax, which induced apoptosis, and BRAF inhibitors, such as trametinib, emerged helping to validate the oncogene addiction hypothesis. There was also a growing recognition that other forms of cell death occur including non‐oncogene addition, which was a product of various forms of cellular stress to which cancer cells were exposed including DNA damage and replicative stress, mitotic stress, proteotoxic stress, metabolic strees and oxidative stress.^18^ Complementary processes that inhibit cell replication include anoikis, which is anchorage loss‐dependent death; necroptosis, which is a caspase‐independent, inflammatory cell death; ferroptosis, which is an iron‐dependent oxidative cell death; and senescence, which is irreversible cell cycle inhibition coupled with the secretion of cytokines, growth factors and proteases.^24^, ^25^ There is considerable interest in finding therapeutics that disrupt many of these processes and the reader is directed to some useful reviews for further details.^26^, ^27^

### 1.3 Nodes and scientific notions

The availability of large annotated cell lines, such as the NCI60 from the National Cancer Institute (see: CellMiner https://discover.nci.nih.gov/cellminer/home.do) or the CCLE from Novartis/Broad Institute (see: https://portals.broadinstitute.org/ccle/), and human tissue samples from the Tumor Cancer Genome Atlas, Dana Farber Cancer Institute, Memorial Sloan Kettering and others, which are now readily addressable via the cBioportal for Cancer Genomics (http://www.cbioportal.org) and other websites, provide investigators a unique ability to rapidly query the mutational status, expression levels, and clinical importance of genes and gene products in specific cancer types. It is important to note that gene mutations or overexpression of a protein even when coupled to poor patient outcomes, does not prove causality; it is only a correlation. Too many cancer pharmacologists have been propelled by such correlations, which has resulted in misguided efforts to identify new therapies.^11^ Remember the ideal drug target should be both necessary and sufficient for the cancer being studied.^11^ The availability of such a large amount of publicly available data, the dramatically lower costs for DNA and RNA sequencing, and pathway analysis programs, such as Reactome (https://reactome.org) or Ingenuity Pathway Analysis (https://analysis.ingenuity.com), have generated numerous computational interaction models, which produce hypotheses about vulnerable signaling nodes of cancer detection intervention. Unfortunately, these big data computationally‐derived models often are not rigorously validated and then fail to live up to their predictions. We tend not to report these failures. Nonetheless, the datasets continue to grow and the computational modeling methodology improves so many investigators hope these big data approaches will uncover valuable and targetable networks for cancer pharmacologists to explore in the future.

In recognition of cancer complexity and tumor dependency on oncogenic mutations, amplifications, and deletions, mechanism agnostic approaches have been adopted to help define cancer vulnerabilities to treatments. Most notable has been the use of synthetic lethal strategies modeled after well‐established genetic methods,^11^ which have facilitated the development of poly(ADP‐ribose) polymerase (PARP) inhibitors for patients whose tumors carry germline mutations that encode the essential DNA repair enzymes *BRCA1* or *BRCA2*.^28^ The availability of gene editing methodologies that disable individual components of the human genome should enable the rapid extension of the synthetic lethal strategy to foster creative drug combinations. Critical for the success of this approach will be identifying drugs that can simulate the loss of a specific gene product and the need for cancer cell specificity.

### 1.4 Immune cancer targets and drugs

Although the role of the tumor microenvironment and stromal cells in cancer progression has always been recognized, many of the prior efforts to therapeutically target the supporting cells, whether they were endothelial cells, pericytes, fibroblasts, or immune cells, were unsuccessful. The long‐term survival benefit, at least for a small number of patients, lead in 2011 to the regulatory approval of ipilimumab, which is a monoclonal antibody targeting the immune suppressive protein CTLA‐4. This profoundly changed the cancer pharmacology landscape. Multiple monoclonal antibodies that block protein‐protein interactions between T cell checkpoint receptors and their cognate ligands have now been approved for clinical use. Immune checkpoint inhibitors, such as pembrolizmab and nivolumab, have become standard of care for multiple cancer types in part because of long‐term remission of a subset of patients. This is illustrated by the overall survival results of the KEYNOTE‐189 trial in patients with previously untreated metastatic nonsquamous non‐small cell lung cancer.^29^ The addition of pembrolizumab to the standard of care combination of a pemetrexed and a platinum‐based drug resulted in longer overall survival than chemotherapy alone. In comparison, a 2002 study examining what at the time were four of the newest cytotoxic chemotherapeutics showed no significant advantage among themselves.^30^ While the two studies should not be directly compared because of fundamental differences in the trial design, for example the former was with previously untreated patients while the later was with pretreated patients, the improved outcomes with the most recent study have generated considerable excitement. Overall, the response rates for single agent immune checkpoint inhibitors in some solid malignancies range from 20% to 40%.^31^ There is almost a complete lack of understanding why tumors in some patients respond and in other patients they do not. Disturbingly, some patients have experienced an accelerated growth rate or hyperprogression of their tumors after single‐agent checkpoint inhibitor treatment with no clear understanding of the causes for this toxicity.^31^ Nonetheless, combinations with immunomodulators and other anticancer agents are being explored intensively. Currently, ClinicalTrials.gov indicates there are 602 active clinical trials with pembrolizumab and 548 with nivolumab. It is difficult to believe this hugely expensive “spray and pray” approach, lacking sound preclinical pharmacological foundations, is scientifically justified.

In addition to the immune checkpoint inhibitors, genetically engineered chimeric antibodies and autologous T cell therapies have emerged. For example, the FDA approved a first‐in‐class bispecific T cell engager (BiTE), blinatumomab, for use in the treatment of B cell acute lymphoblastic leukemia. The BiTE comprise two joined monoclonal antibodies; one end of the BiTE binds to a molecule on T cells, and the other end binds to CD19 on surface of acute lymphoblastic leukemia cells, facilitating cancer cell death. In April 2017, the first chimeric antigen receptor T cell therapy, tisagenleclucel, was approved by the FDA for acute lymphoblastic leukemia.

### 1.5 The future of cancer pharmacology

With cancer pharmacology, futuristic predictions are almost always inaccurate, if not outright wrong. Nonetheless, it seems likely that drug resistance will remain a significant problem irrespective of the therapeutic modality employed. For immune‐oncology to advance, a more comprehensive understanding the molecular and cellular factors that determine response and resistance will be essential. With the ascendency of biologics, such as antibodies, and cellular therapies, such as the chimeric antigen receptor T‐cell tisagenlecleucel, perhaps this is the dénouement of small molecules for cancer therapy? It is interesting to entertain the possibility of a time when orally available small molecules, dominated by the skills of medicinal chemists, are in the minority of the therapeutic armamentarium of the oncologists and hematologists.

With the emphasis on precision medicine and target therapies, we have begun to see creative so‐called “basket” or “umbrella” cancer drug clinical trials and even regulatory approvals of drugs targeting the presence of a specific genetic alteration that are not linked to a particular anatomical tumor site. This is occurring with PARP inhibitors and with immune‐oncology drugs. Further information about these trial designs can be found elsewhere.^32^

A large fraction of the appealing oncoproteins and tumor suppressors remain under the cloud of being undrugged, including transcription factors like Myc, intracellular signaling participants like Ras, and protein tyrosine phosphatases.^10^ These may be addressable with the emergence of chemical degraders, often referred to as proteolysis‐targeting chimeras or PROTACs.^33^ The first of these proximity‐induced bifunctional somewhat small molecules that targets the androgen receptor for degradation by an E3 ubiquitin ligase for prostate cancer, namely ARV‐110, has entered Phase 1 clinical trials. There is considerable enthusiasm for the use of chemical degraders for many other intracellular cancer targets that heretofore have not been viewed as druggable^33^ but it remains to be seen if this general approach will be limited because of poor anatomical distribution issues, such as penetrating the blood‐brain barrier, or significant unanticipated untoward effects. There is also renewed interest in the use of modified nucleic acid‐based therapies, including microRNA.^34^ There have been exciting preclinical results reported using endosomal delivery systems as a platform to enhance the delivery and efficacy of short interfering RNA or short hairpin RNA specific to oncogenic KRas^G12D^, a common mutation in pancreatic cancer.^35^ Nanovehicles are being explored that may be able to efficiently carry and deliver anticancer agents to tumor sites, exploiting differences between normal tissue and tumor microenvironments, such as vascular abnormalities, hypoxia and acidic pH.^36^ I would like to believe we will see some consolidation around the optimal in vitro and in vivo model test systems for new agents and combinations in the future.

In summary, if this is not the golden age of cancer pharmacology, it most certainly is a different era. The explosion in new therapies, the possibilities of numerous possible combinations, and the persistence of drug resistance cries out for more involvement of cancer pharmacologists. The rich emerging omics databases may provide clues as to how to best use the next generation of cancer therapies and how to avoid resistance. The linkage of diagnostics, such as the estrogen and progesterone receptor status, with drug treatment regimens also has become routine. In many cases cancer is becoming a chronic disease with long term treatment strategies similar to other nonmalignant diseases. Concerns about the “financial toxicity” of new cancer agents, especially in the United States where new drug costs are not regulated and exceedingly high, and the low success rate of cancer clinical trials (<10% of the anticancer drugs that enter phase 1 trials are ever approved for clinical use), however, could weigh heavy on how we proceed. The sobering commentaries by others^6^, ^37^, ^38^ on the high costs of drugs and the poor outcomes of cancer clinical trials are worth the reader's attention.

## ETHICS STATEMENT

All statements in this article were constructed with the appropriate ethical considerations.

## DISCLOSURES

The author (JSL) is Chief Scientific Officer of KeViRx, Inc, which is developing novel anticancer small molecules, but declares no conflict of interest.

## AUTHOR CONTRIBUTIONS

The author (JSL) wrote the manuscript, generated the figures, and is responsible for its contents.

## DATA REPOSITORY LINK

A data repository link is not applicable to the contents of this manuscript.

## References

[1] Siegel RL , Miller KD , Jemal A . Cancer Statistics, 2018. CA: A Cancer J Clinicians 2018; 68:7‐30. 10.3322/caac.21442 29313949

[2] Bray F , Ferlay J , Soerjomataram I , Siegel RL , Torre LA , Jemal A . Global cancer statistics 2018: GLOBOCAN estimates of incidence and mortality worldwide for 36 cancers in 185 countries. CA A Cancer J Clin. 2018;68:394‐424. 10.3322/caac.21492 30207593

[3] Hickman JA , Lazo JS . Changes and challenges–the world post‐Gleevec (Glivec). Curr Opin Pharmacol. 2002;2:357‐360. 12127866 10.1016/s1471-4892(02)00187-x

[4] Hanahan D , Weinberg RA . The hallmarks of cancer. Cell 2000;100:57‐70. 10647931 10.1016/s0092-8674(00)81683-9

[5] Prasad V , Fojo T , Brada M . Precision oncology: origins, optimism, and potential. Lancet Oncol. 2016;17:e81‐e86. 26868357 10.1016/S1470-2045(15)00620-8

[6] Tannock IF , Hickman JA . Limits to personalized cancer medicine. NEJM. 2016;375:1289‐1294. 27682039 10.1056/NEJMsb1607705

[7] Ashley EA . Towards precision medicine. Nature Rev Genet. 2016;17:507‐522. 27528417 10.1038/nrg.2016.86

[8] Hyman DM , Taylor BS , Baselga J . Implementing genome‐driven oncology. Cell 2017;168:584‐599. 28187282 10.1016/j.cell.2016.12.015 PMC5463457

[9] Sun J , Wei Q , Zhou Y , Wang J , Liu Q , Xu H . A systematic analysis of FDA‐approved anticancer drugs. BMC Syst Biol. 2017;11:87. 28984210 10.1186/s12918-017-0464-7 PMC5629554

[10] Lazo JS , Sharlow ER . Drugging undruggable molecular cancer targets. Ann Rev Pharmacol Toxicol. 2015;56:23‐40. 26527069 10.1146/annurev-pharmtox-010715-103440

[11] Kaelin WG Jr . Common pitfalls in preclinical cancer target validation. Nature Rev Cancer. 2017;545:387. 10.1038/nrc.2017.32 28524181

[12] Wood LD , Parsons DW , Jones S , et al. The genomic landscapes of human breast and colorectal cancers. Science 2007;318:1108‐1113. 17932254 10.1126/science.1145720

[13] Nath S , Devi GR . Three‐dimensional culture systems in cancer research: Focus on tumor spheroid model. Pharmacol Therap. 2016;163:94‐108. 27063403 10.1016/j.pharmthera.2016.03.013 PMC4961208

[14] Weeber F , Ooft SN , Dijkstra KK , Voest EE . Tumor organoids as a pre‐clinical cancer model for drug discovery. Cell Chem Biol. 2017;24:1092‐1100. 28757181 10.1016/j.chembiol.2017.06.012

[15] Shoemaker RH . The NCI60 human tumour cell line anticancer drug screen. Nat Rev Cancer 2006;6:813‐823. 16990858 10.1038/nrc1951

[16] Brooks MD , Burness ML , Wicha MS . Therapeutic implications of cellular heterogeneity and plasticity in breast cancer. Cell Stem Cell 2015;17:260‐271. 26340526 10.1016/j.stem.2015.08.014 PMC4560840

[17] Hanahan D , Weinberg RA . Hallmarks of cancer: the next generation. Cell 2011;144:646‐674. 21376230 10.1016/j.cell.2011.02.013

[18] Luo J , Solimini NL , Elledge SJ . Principles of cancer therapy: oncogene and non‐oncogene addiction. Cell 2009;136:823‐837. 19269363 10.1016/j.cell.2009.02.024 PMC2894612

[19] Gengenbacher N , Singhal M , Augustin HG . Preclinical mouse solid tumour models: status quo, challenges and perspectives. Nature Rev Cancer 2017;17:751‐765. 29077691 10.1038/nrc.2017.92

[20] Day CP , Merlino G , Van Dyke T . Preclinical mouse cancer models: a maze of opportunities and challenges. Cell 2015;163:39‐53. 26406370 10.1016/j.cell.2015.08.068 PMC4583714

[21] Guo S , Jiang X , Mao B , Li QX . The design, analysis and application of mouse clinical trials in oncology drug development. BMC Cancer 2019;19:718. 31331301 10.1186/s12885-019-5907-7 PMC6643318

[22] Wartha K , Herting F , Hasmann M . Fit‐for purpose use of mouse models to improve predictivity of cancer therapeutics evaluation. Pharmacol Therap. 2014;142:351‐361. 24412280 10.1016/j.pharmthera.2014.01.001

[23] Hickman JA , Potten CS , Merritt AJ , Fisher TC . Apoptosis and cancer chemotherapy. Phil Trans Royal Soc London Series B, Biol Sci. 1994;345:319‐325. 10.1098/rstb.1994.0112 7846129

[24] Ashrafizadeh M , Mohammadinejad R , Tavakol S , Ahmadi Z , Roomiani S , Katebi M . Autophagy, anoikis, ferroptosis, necroptosis, and endoplasmic reticulum stress: potential applications in melanoma therapy. J Cell Physiol. 2019;234:19471‐19479. 31032940 10.1002/jcp.28740

[25] Collado M , Serrano M . Senescence in tumours: evidence from mice and humans. Nature Rev Cancer 2010;10:51‐57. 20029423 10.1038/nrc2772 PMC3672965

[26] Angeli J , Shah R , Pratt DA , Conrad M . Ferroptosis inhibition: mechanisms and opportunities. Trends Pharmacol Sci. 2017;38:489‐498. 28363764 10.1016/j.tips.2017.02.005

[27] Short S , Fielder E , Miwa S , von Zglinicki T . Senolytics and senostatics as adjuvant tumour therapy. EBioMedicine 2019;41:683‐692. 30737084 10.1016/j.ebiom.2019.01.056 PMC6441870

[28] Lord CJ , Ashworth A . PARP inhibitors: synthetic lethality in the clinic. Science 2017;355:1152‐1158. 28302823 10.1126/science.aam7344 PMC6175050

[29] Gandhi L , Rodríguez‐Abreu D , Gadgeel S , et al. Pembrolizumab plus chemotherapy in metastatic non‐small‐cell lung cancer. N Engl J Med 2018;378:2078‐2092. 29658856 10.1056/NEJMoa1801005

[30] Schiller JH , Harrington D , Belani CP , et al. Comparison of four chemotherapy regimens for advanced non‐small‐cell lung cancer. N Eng J Med. 2002;346:92‐98. 10.1056/NEJMoa011954 11784875

[31] Kato S , Goodman A , Walavalkar V , Barkauskas DA , Sharabi A , Kurzrock R . Hyperprogressors after immunotherapy: analysis of genomic alterations associated with accelerated growth rate. Clin Cancer Res. 2017;23:4242‐4250. 28351930 10.1158/1078-0432.CCR-16-3133 PMC5647162

[32] Simon R . Critical review of umbrella, basket, and platform designs for oncology clinical trials. Clin Pharmacol Therap. 2017;102:934‐941. 28795401 10.1002/cpt.814

[33] Neklesa TK , Winkler JD , Crews CM . Targeted protein degradation by PROTACs. Pharmacol Therap. 2017;174:138‐144. 28223226 10.1016/j.pharmthera.2017.02.027

[34] Rupaimoole R , Slack FJ . MicroRNA therapeutics: towards a new era for the management of cancer and other diseases. Nat Rev Drug Discov. 2017;16:203‐222. 28209991 10.1038/nrd.2016.246

[35] Kamerkar S , LeBleu VS , Sugimoto H , et al. Exosomes facilitate therapeutic targeting of oncogenic KRAS in pancreatic cancer. Nature 2017;546:498‐503. 28607485 10.1038/nature22341 PMC5538883

[36] Dai Y , Xu C , Sun X , Chen X . Nanoparticle design strategies for enhanced anticancer therapy by exploiting the tumour microenvironment. Chem Soc Rev. 2017;46:3830‐3852. 28516983 10.1039/c6cs00592f PMC5521825

[37] Burotto M , Wilkerson J , Stein WD , Bates SE , Fojo T . Adjuvant and neoadjuvant cancer therapies: a historical review and a rational approach to understand outcomes. Sem Oncol. 2019;46:83‐99. 10.1053/j.seminoncol.2019.01.002 30738604

[38] Fojo T . The high cost of ignorance in oncology. Sem Oncol. 2016;43:623‐624. 10.1053/j.seminoncol.2016.11.010 28061979

